# Infrastructure, logistics and clinical practice management of acute trauma hemorrhage and coagulopathy: a survey across German trauma centers

**DOI:** 10.1007/s00068-021-01788-9

**Published:** 2021-09-26

**Authors:** Vivien Karl, Nadine Schäfer, Marc Maegele

**Affiliations:** 1 Department of Medicine, Faculty of Health, Institute for Research in Operative Medicine (IFOM), Witten/Herdecke University, Ostmerheimer Str. 200, Building 38, 51109 Cologne, Germany; 2 Department of Traumatology, Orthopedic Surgery and Sports Traumatology, Cologne-Merheim Medical Center (CMMC), Witten/Herdecke University, Campus Cologne-Merheim, Ostmerheimer Str. 200, 51109 Cologne, Germany

**Keywords:** Trauma hemorrhage, Coagulopathy, Treatment, Guidelines

## Abstract

**Purpose:**

Early detection and management of acute trauma hemorrhage and coagulopathy have been associated with improved outcomes, but local infrastructure, logistics and clinical strategies may differ.

**Methods:**

To assess local differences in infrastructure, logistics and clinical management of acute trauma hemorrhage and coagulopathy we have conducted a web-based survey amongst clinicians working in DGU^®^-certified supraregional, regional and local trauma centers.

**Results:**

137/1875 respondents completed the questionnaire yielding a response rate of 7.3%. The majority specified to work as head of department or senior consultant (95%) in trauma/orthopedic surgery (80%) of supraregional (38%), regional (34%) or local (27%) trauma centers. Conventional coagulation assays are most frequently used to monitor bleeding trauma patients. Only half of the respondents (53%) rely on extended coagulation tests, e.g. viscoelastic hemostatic assays. Tests to assess preinjury use of direct oral anticoagulants and platelet inhibitors are still not widely available and vary according to level of care. Conventional blood products are widely available but there remain differences between trauma centers of different level of care to access other hemostatic therapies, e.g. coagulation factor concentrates. Trauma centers of higher level of care are more likely to implement treatment protocols.

**Conclusion:**

This survey confirms still existing differences in infrastructure, logistics and clinical practice management for the detection of acute trauma hemorrhage and coagulopathy amongst DGU^®^-certified supraregional, regional and local trauma centers. Further work is recommended to locally implement diagnostics, therapies and treatment algorithms compliant to current guidelines to ensure the best possible outcomes in bleeding trauma patients.

**Supplementary Information:**

The online version contains supplementary material available at 10.1007/s00068-021-01788-9.

## Introduction

Trauma still ranges among the most common causes of death worldwide with death from uncontrolled hemorrhage and failure to restore hemostasis as the most common cause of preventable death after trauma [1, 2]. One in three to four patients with severe injury and bleeding displays both hemodynamic and laboratory blood clotting abnormalities upon Emergency Department (ED) arrival [3–5]. Vice versa, early diagnosis and aggressive management with the overall aim to rapidly stop and control further blood loss and to restore hemostasis can significantly improve outcomes in terms of both morbidity and mortality [2, 6–8]. In the military setting, mortality among the most critically injured casualties during the recent conflicts in Iraq and Afghanistan (2001–2017) was reduced by 44% and this was linked to three key interventions, e.g. (1) the early use of tourniquets, (2) prehospital transport within 60 min, and (3) the early access to blood products and hemostatic therapies [7].

The European guideline on management of major bleeding and coagulopathy following trauma provides a series of evidence-based recommendations on how to approach critically injured and bleeding trauma patients in terms of early diagnostics, surgical and hemostatic management and implementation of treatment algorithms [9]. The adherence to these evidence-based treatment algorithms has frequently been associated with improved survival [10–12]. However, huge variability in the care for bleeding trauma patients including hemostatic control still exists, even among major trauma centers, mostly due to differences in infrastructure, logistics and clinical practice [13]. We have conducted a web-based survey across German trauma centers to assess local differences in infrastructure, logistics and clinical practice management of acute trauma-hemorrhage and coagulopathy. Specific focus was given to the preinjury use anticoagulants and antiplatelet agents. The results were compared to a similar survey conducted by our group in 2014 for changes in the early access to advanced diagnostics, blood products and hemostatic agents and clinical management over time [14].

## Methods

A web-based survey was designed and programmed with accreditation of the IT division (BIT, Bereich Informationstechnologie) of the Witten/Herdecke University using LimeSurvey (Version 3.26.0 + 210,419), an open source application (https://www.limesurvey.org/). Overall, the questionnaire consisted of seventeen questions: nine single answer questions, seven multiple answer questions and one table with four options for selection. The original questionnaire is provided in the Online Resource 1. According to the answer, the questionnaire guided to the next relevant question, leading to a minimum number of thirteen questions. The response to these thirteen questions was mandatory.

In detail, the questions one and two focused on the level of experience and medical specialization of the respondents. The questions three to five captured information concerning the level of care, trauma load and estimated percentage of patients with hemostatic disorders admitted to the respective trauma centers the respondents worked in. The questions six and seven aimed to assess the respondents’ attitude and concerns towards the preinjury use of different anticoagulants and antiplatelet agents including the novel direct oral anticoagulants (DOACs) and platelet inhibitors (PIs). The next five questions focused on measures and strategies to assess and control bleeding in trauma patients with coagulopathy in the respective centers. This included both diagnostics and therapeutic strategies to rapidly control and stop bleeding and to restore hemostasis with blood products, coagulation factor concentrates and hemostatic agents. Questions thirteen to sixteen addressed the use and adherence to evidence-based treatment algorithms. Lastly, the respondents were asked for specific measures and actions that should be considered for local improvement of care and the management for bleeding trauma patients in general.

The study was planned to find a ± 5% precision around a prevalence of 10% (95% confidence interval). This would require 150 responses. With an estimated response rate of 10% at least 1500 questionnaires needed to be sent out. On March 2nd, 2021, the questionnaire was distributed through the Academy of Trauma (Akademie der Unfallchirurgie (AUC); Munich) to 1875 clinicians working in DGU^®^-certified supraregional, regional and local trauma centers within their respective local Trauma Networks (Traumanetzwerke DGU^®^) either as administrators or deputies for the German TraumaRegister DGU^®^ (TR-DGU^®^). The Whitebook of the German Society for Trauma Surgery (DGU^®^) provides recommendations on structure, organization, installations and equipment to promote quality, safety and reliability in the medical care of the severely injured in Germany [15]. In accordance, hospital infrastructure has been divided into three care categories that have been catalogued according to special structure and process criteria and given codes, e.g. (1) local trauma centers, (2) regional trauma centers, and (3) supraregional trauma centers, with vertical patient flow between the centers according to injury severity and complexity within the Trauma Networks (Traumanetzwerke DGU^®^) [15]. Regular training including refresher courses according to the Advanced Trauma Life Support (ATLS^®^) format are mandatory for in-hospital staff of certified trauma centers and resuscitation principles in certified trauma centers should follow evidence-based guidelines, e.g. the official multidisciplinary S3-guideline on “Polytrauma” and/or the “European guideline of the management of bleeding following major trauma”[9, 15, 16].

The AUC is closely affiliated with the national German Society for Trauma Surgery (DGU^®^) and responsible for the development and activity of networks and structures. The AUC supports the Trauma Networks (Traumanetzwerke DGU^®^)through exchange programs, continuous education and organizes and hosts training programs, e.g. Advanced Trauma Life Support (ATLS^®^) or Prehospital Trauma Life Support (PHTLS^®^). The AUC functions as data holder for the German Trauma Register database. For quality control, all trauma centers that participate in acute trauma care throughout Germany need to be certified and need to enter their local data, either as basic dataset or extended documentation, on local practice and outcome into the TR-DGU^®^ database. By this policy, the data entered into the TR-DGU^®^ may be considered representative for trauma care in Germany. Two weeks after the questionnaire was distributed, an email reminder was sent out. The survey was closed after three weeks on March 23rd, 2021.

The collected data were analyzed using LimeSurvey application and the embedded data export function of SPSS (IBM SPSS Statistics, Version 26). Figures were created using Microsoft PowerPoint (Powerpoint 2016). The survey was approved by the Witten/Herdecke University Committee of Ethics (No. 235/2020).

## Results

Out of 1875 questionnaires send out, 173 were returned and 137 were returned completed, yielding an overall response rate of 7.3%. The majority of the respondents worked as either head of department or senior consultant (130/137; 95%) predominantly specialized in trauma and orthopedic surgery (109/137; 80%). 52/137 (38%) respondents declared to work in supraregional trauma centers, e.g. academic (19/137; 14%) and non-academic (33/137; 24%), 46/137 (34%) in regional trauma centers, 37/137 (27%) in local trauma centers and 2/137 (1%) in non-certified trauma centers, respectively. Half of the respondents (68/137; 50%) estimated the annual trauma load to be admitted to their local centers at over 25 patients with an Injury Severity Score ≥ 16 while 24/137 (18%) declared to receive 100 cases or more per year in their respective centers. More than half of the respondents (72/137; 53%) estimated the percentage of bleeding and coagulopathic trauma patients in need for hemostatic therapies at their local center to range between 10 and 30%. Trauma management including coagulation management in the respective centers is mainly interdisciplinary with trauma/orthopedic surgery being the responsible specialty in 123/137 (90%) of settings followed by anesthesiology (105/137; 77%) and intensive care (40/137; 29%).

### Diagnostic approaches

Almost all respondents, independent of background and hospital level of care, stated to have access to the results from routine laboratory testing for hemoglobin, hematocrit, pH and base excess (BE) within 30 min of patient arrival to the ED. Results for conventional coagulation assays (CCAs), e.g. prothrombin time (PT), international normalized ratio (INR), Quick’s value and activated partial thromboplastin time (aPTT), are available within 30 min in over 70% (103/137) of the centers and within 30 to 60 min in 34/137 (25%) centers, respectively. 118/137 (86%) respondents reported to have access to platelet counts within 30 min of patient arrival to the ED while only half of the respondents (72/137; 53%) declared to assess platelet function.

**Fig. 1:**
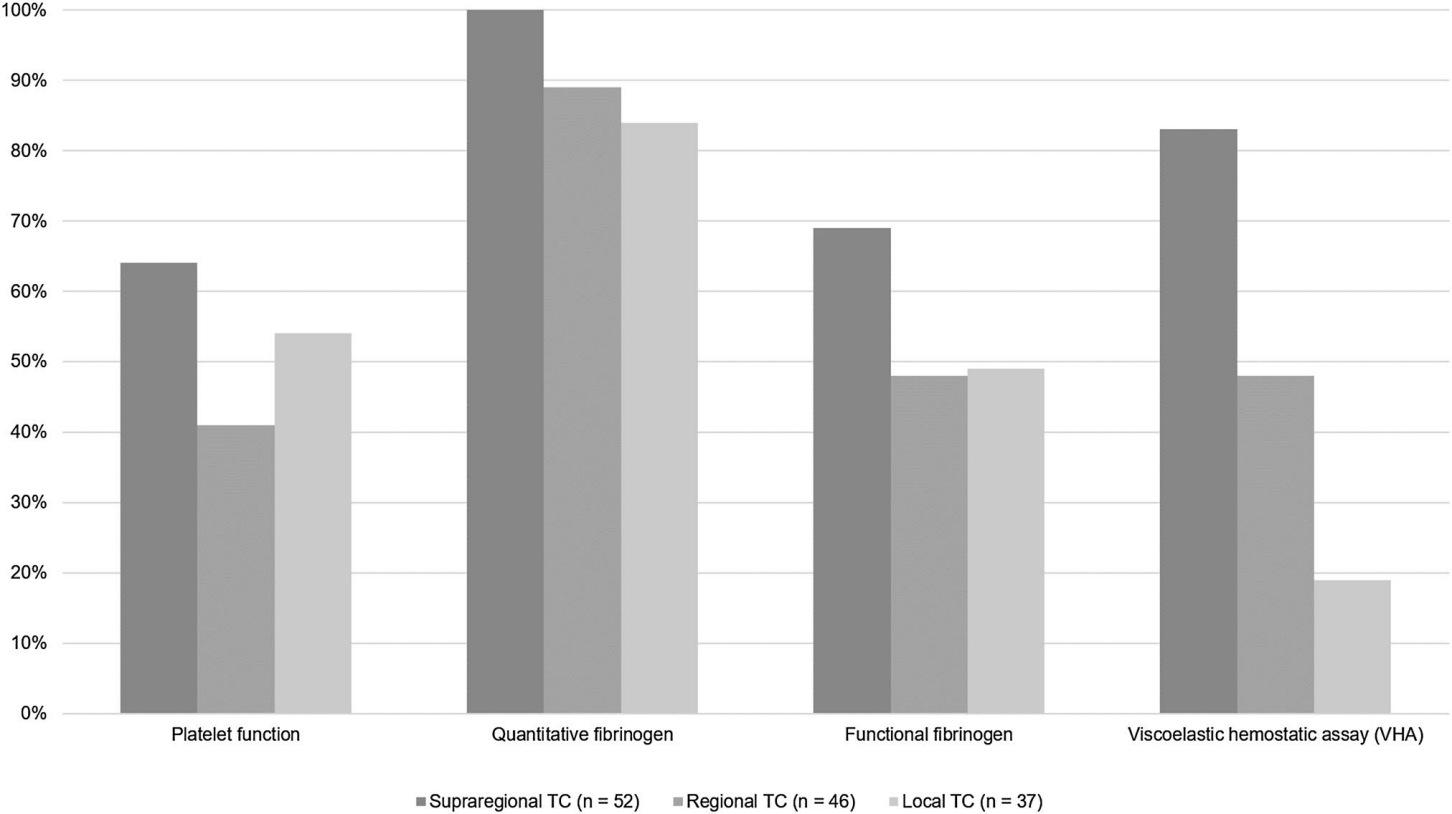
Platelet function and extended coagulation diagnostics across different levels of care provided by the centers (*TC* = trauma center; *n* = number of respondents)

The use of advanced coagulation testing increases with higher level of care provided by the respective trauma centers (Fig. 1). Approximately half of the respondents (72/137; 53%) declared to have access to functional viscoelastic hemostatic assays (VHAs, e.g. thromboelastography (TEG) or rotational thromboelastometry (ROTEM)), with results available within 30 min in 41/137 (30%) centers, within 30 to 60 min in 16/137 (12%) centers, and beyond 60 min in 15/137 (11%) centers, respectively. The remaining 65/137 (48%) respondents stated non-availability or non-use of VHAs in their local setting.

Platelet function testing was declared as least used and this even in supraregional trauma centers (Fig. 1). Only 72/137 (52%) respondents declared to have access to platelet function tests, with results from platelet aggregometry being available within 60 min in only 45/137 (33%) of settings. 55/137 (40%) respondents declared to have access to the results from quantitative fibrinogen measurements, e.g. according to Clauss, within 30 min of arrival, and approximately the same number (50/137; 37%) within 30 to 60 min of patient arrival to the ED, respectively.

### Assessment of preinjury use of anticoagulants and antiplatelet agents

**Fig. 2:**
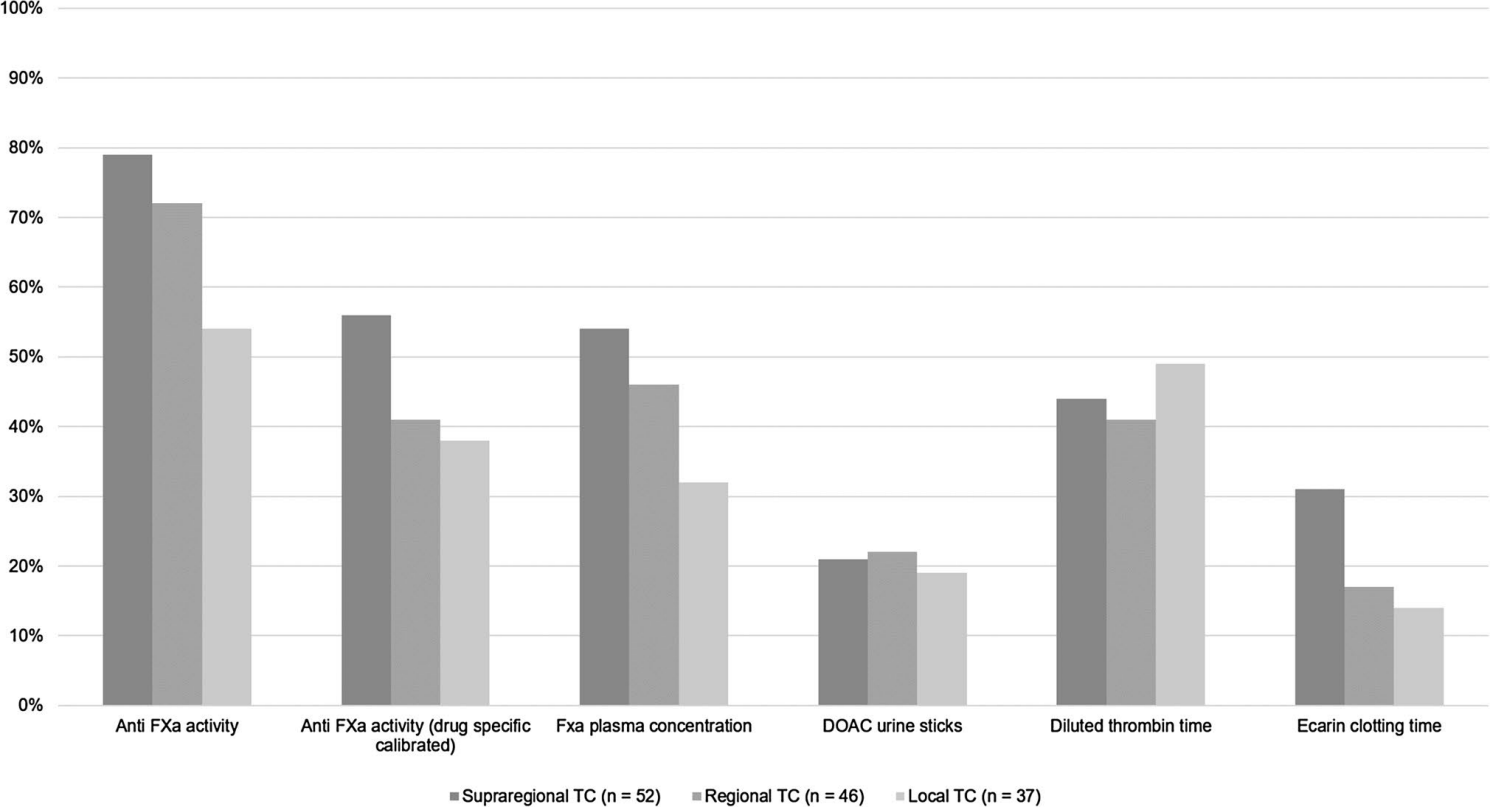
DOAC-specific testing methods according to levels of care (*TC* = trauma center; *n* = number of respondents)

The availability and the use of laboratory tests for anti-factor Xa (FXa) activity, drug specific anti-FXa activity, FXa plasma concentration and ecarin clotting time (ECT) declines with lower level of care provided by the respective trauma centers (Fig. 2). 95/137 (69%) respondents declared to assess anti FXa activity with results available within 30 min of patient arrival to the ED (10/137; 7%), within 30 to 60 min (33/137; 24%) and beyond 60 min (52/137; 38%), respectively. 42/137 (31%) respondents reported non-availability or non-use of this assessment. Calibrated drug specific FXa tests were reported less used by only 63/137 (46%) respondents. Comparable responses were obtained for the use of DOAC plasma concentration level measurements. Only 19/137 (14%) respondents declared to have access to the results within 60 min of patient arrival to the ED and 75/137 (55%) declared non-availability or non-use of this assessment in their local setting. Less than half of the respondents (61/137; 44%) stated the use of diluted thrombin time (dTT) with results being available within 30 min (21/137; 15%), within 30 to 60 min (21/137; 15%) and beyond 60 min (19/137; 14%) of patient arrival to the ED. Similarly, only 29/137 (21%) respondents declared the use of ECT with results being available within 30 min (4/137; 3%), within 30 to 60 min (6/137; 4%) and beyond 60 min (19/137; 14%) of patient arrival in the ED, respectively. 108/137 (79%) respondents declared non-availability or non-use of this measurement in their local setting. One out of five respondents (28/137; 20%) declared the use of DOAC urine sticks.

### Therapy

**Fig. 3:**
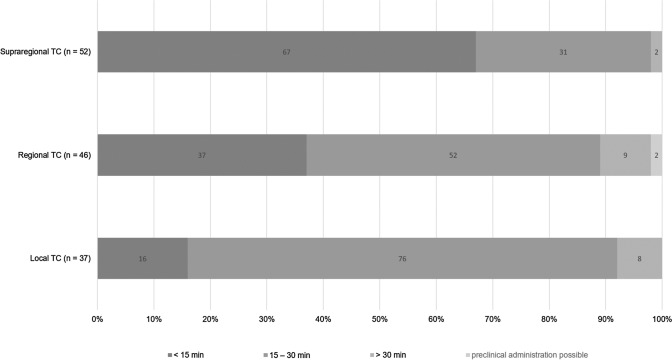
Time to administration of the first blood product according to levels of care (*TC* = trauma center; *n* = number of respondents)

**Fig. 4:**
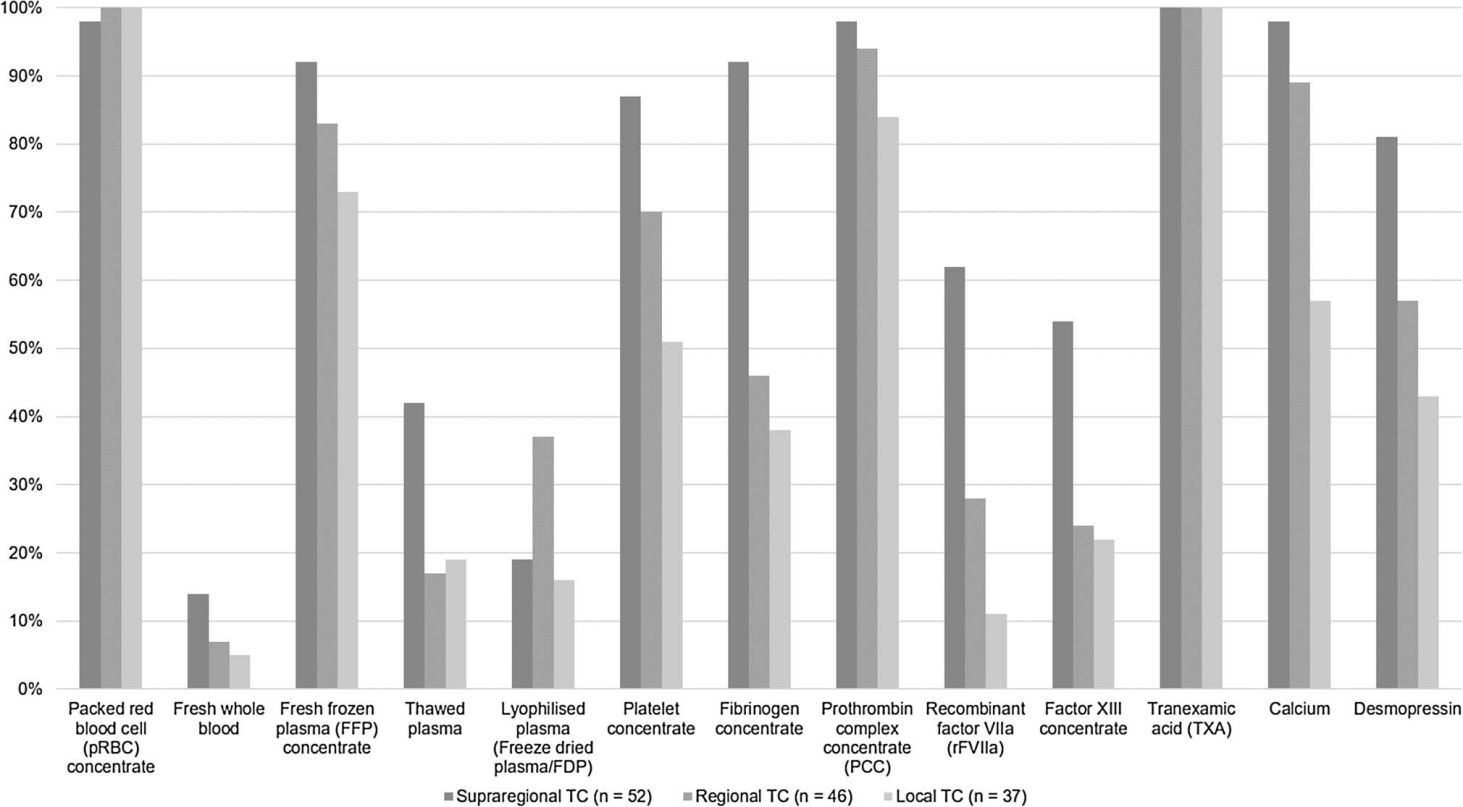
Access to blood products and hemostatic agents according to levels of care (*TC* = trauma center; *n* = number of respondents)

Overall, 128/137 (93%) respondents declared to be able to deliver blood products within 30 min of patient arrival to the ED; 58/137 (42%) within 15 min, respectively. However, the time window to initiate blood product administration differs between the levels of care provided by the centers (Fig. 3). All respondents, independent of background and level of care, stated to have access to packed red blood cell concentrates (pRBC) and tranexamic acid (TXA) in their local setting. Figure 4 shows the differences between supraregional, regional and local trauma centers with respect to access to other blood products and hemostatic agents including coagulation factor concentrates. While more than 90% (126/137) of the respondents working in supraregional trauma centers declared to have access to fibrinogen concentrates, this was only the case in 46% (63/137) of the respondents from regional, and in only 38% (52/137) of the respondents from local trauma centers, respectively. Similar differences according to level of care were reported for other coagulation factor concentrates, e.g. recombinant factor VIIa (rFVIIa), factor XIII, but also for platelet concentrates (Fig. 4). Interestingly, almost 40% (51/137) of the respondents from regional trauma centers declared to work with freeze-dried plasma (Fig. 4).

**Fig. 5:**
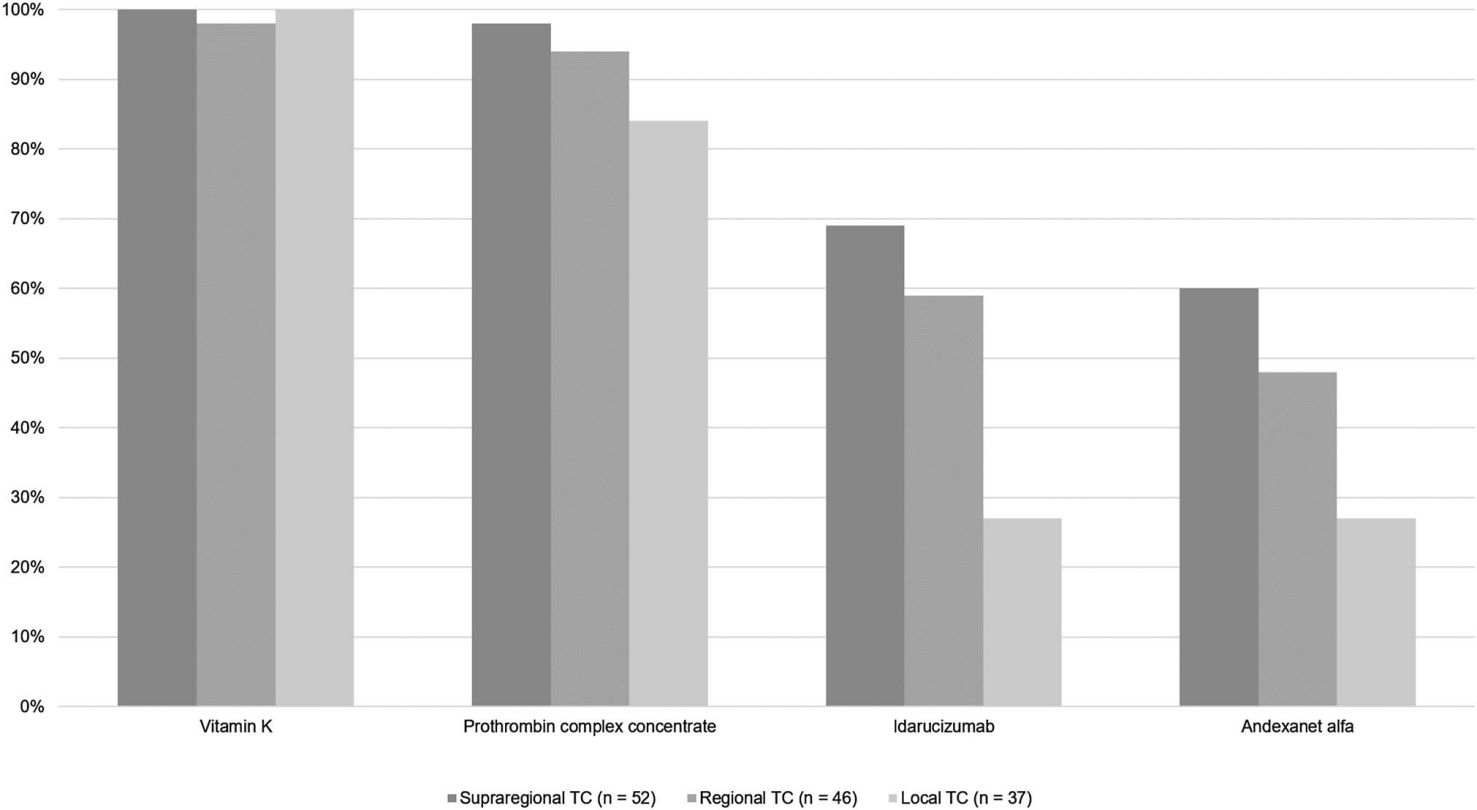
Access to vitamin K antagonists vs. DOAC antidotes according to levels of care (*TC* = trauma center; *n* = number of respondents)

Almost all respondents (133/137; 97%) considered the preinjury use of DOACs and antiplatelet agents as a potential threat to bleeding trauma patients with 78/137 (57%) declaring major concerns with these drugs in the emergency setting. On drug level, these concerns were mainly expressed against FXa inhibitors (112/133; 82%), direct thrombin inhibitors (85/133; 62%), vitamin K antagonists (58/133; 42%) and PY212 antagonists (55/133; 40%). Almost all respondents, independent of background and level of care, declared to have access to reversal agents for vitamin K antagonists, e.g. vitamin K and prothrombin complex concentrate (PCC) (Fig. 5). In contrast, the access to specific antidotes for the emergency reversal of DOACs, e.g. idarucizumab and andexanet alfa, was reported as much less and this across all levels of care. On average, one in three respondents even from supraregional trauma centers declared non-availability or non-access to neither drug in their local setting (Fig. 5).

**Fig. 6:**
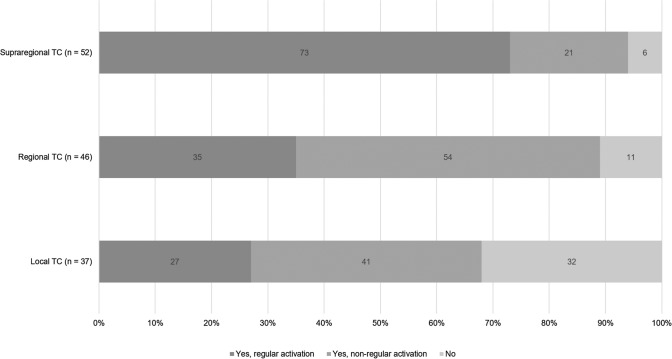
Implementation of a treatment algorithm according to levels of care (*TC* = trauma center; *n* = number of respondents)

**Fig. 7:**
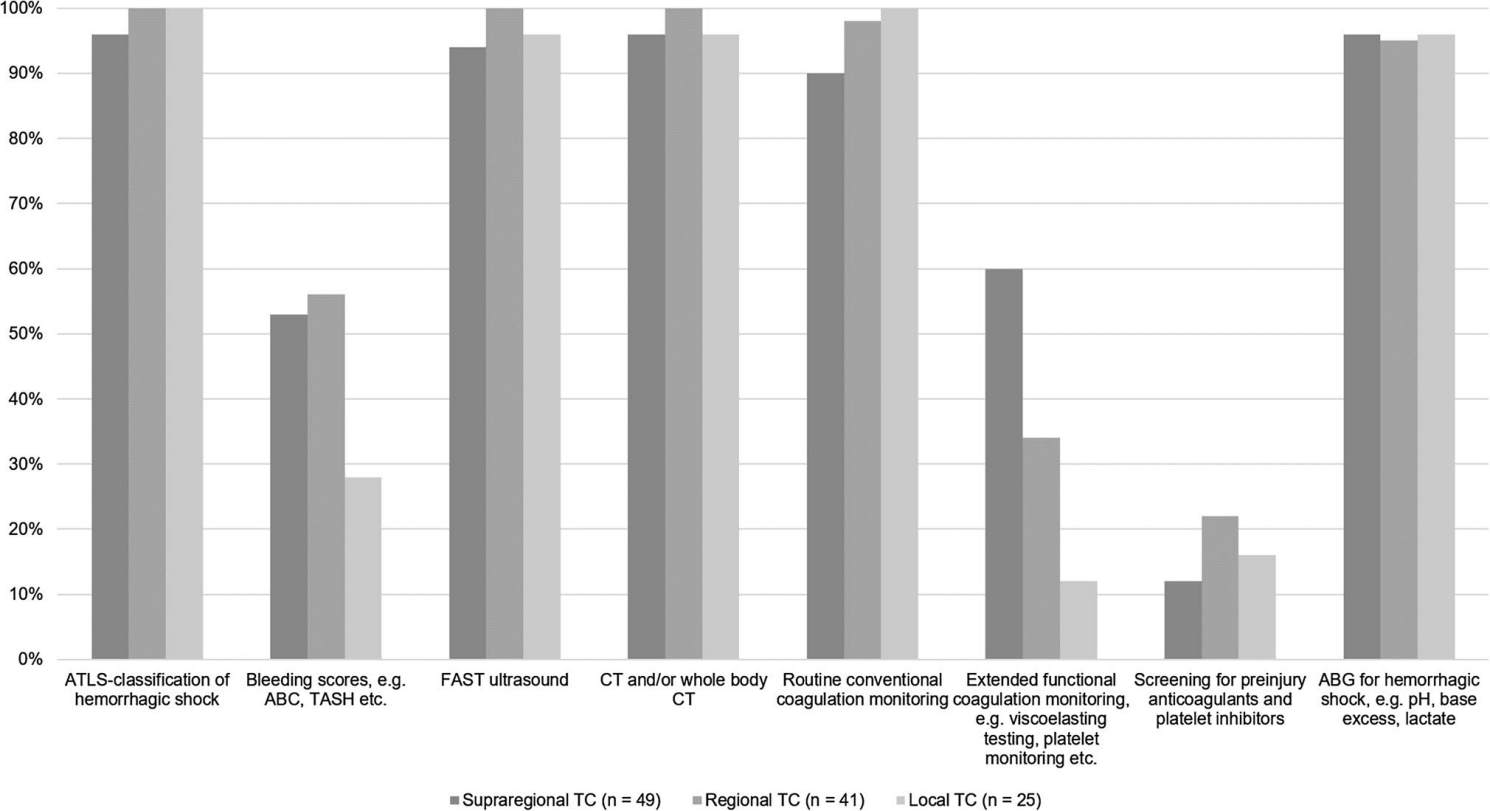
Rationale behind treatment algorithms for assessment, investigation and monitoring (*TC* = trauma center; *n* = number of respondents)

**Fig. 8:**
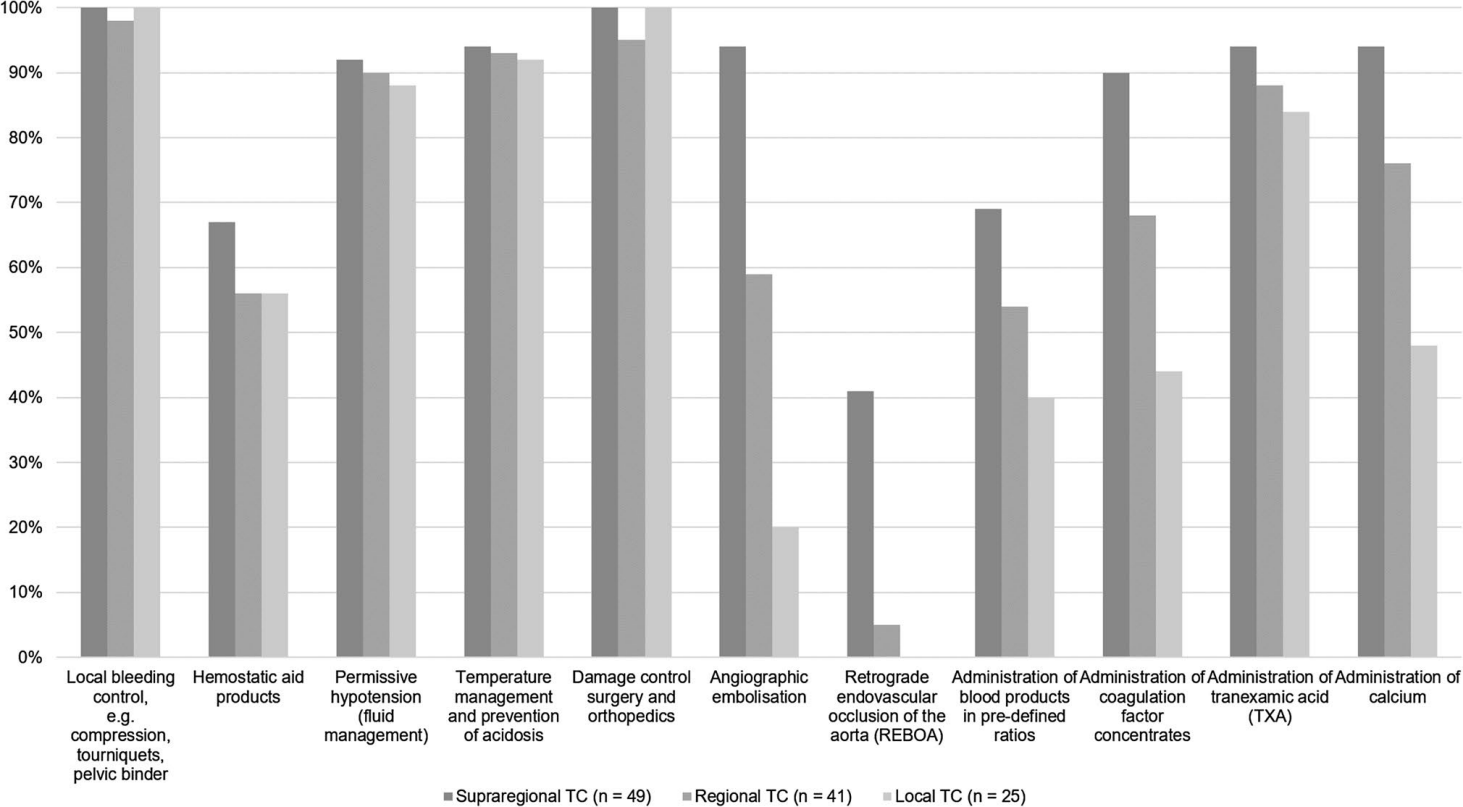
Rationale behind treatment algorithms for immediate intervention (*TC* = trauma center; *n* = number of respondents)

For the management of bleeding trauma patients, 115/137 (84%) respondents stated to follow a treatment algorithm, e.g. a massive transfusion protocol. However, 51/137 (37%) respondents admitted inconsistent or non-regular use. While over 90% (49/52) of the respondents from supraregional trauma centers declared either regular or non-regular use of a treatment algorithm for bleeding trauma patients, one in three respondents from local trauma centers declared absence of a treatment algorithm in their local setting (Fig. 6). As underlying rationale for local treatment algorithms, 64/115 (47%) respondents specified international guidelines, while 82/115 (60%) specified national guidelines developed by national societies and/or health authorities. 65/115 (47%) respondents additionally declared to use the pertinent literature and results from clinical trials to inform their local guidelines. Major aspects covered by local guidelines with differences between centers according to level of care are summarized in Figs. 7 and 8.

Table 1 provides a synopsis of responses independent of background and level of care for improvements in the care for the severely injured and bleeding patient with coagulopathy on local level. The vast majority of the respondents stressed the need for better timely assessment of patients on preinjury anticoagulant and antiplatelet therapies and for access to specific antidotes.

**Table 1:** “Wishlist” to improve local practices in detecting and managing severe bleeding and coagulopathy of trauma patients

	% (*n* = 137)
Treatment algorithms for the management of bleeding and coagulopathy after trauma	45
Checklists to guide clinical practice	42
Faster turn-around times for standard coagulation assays	32
Advanced technology for early detection and monitoring of severe bleeding/coagulopathy	44
Tests to detect patients under influence of DOACs and platelet aggregation	64
Fast availability of blood products	22
Fast availability pf additional agents/drugs to support coagulation function	21
Fast availability of antidotes against DOACs	61
Interdisciplinary training programs to improve clinical skills related on the early detection and treatment of bleeding and coagulopathy after trauma	50
Improved strategies for faster vertical patient transfers to trauma centers of higher level of care	20
No need for improvement	2

## Discussion

The present survey was initiated to reevaluate the current state and the potential progress in the care for the severely injured and bleeding trauma patient with coagulopathy across German trauma centers providing different levels of care. The response rate of 7.3% was similar to our own previous survey and to that of our colleagues both conducted back in 2014 [14, 17]. The present survey was distributed through the AUC to clinicians working in DGU^®^-certified trauma centers affiliated to the German TraumaRegister (TR-DGU^®^). The vast majority of the respondents were senior clinicians working either in supraregional or regional trauma centers and we conclude that that this survey is therefore supported by strong clinical experience.

Although the survey was distributed, by nature, mainly among clinicians specialized in trauma and orthopedic surgery (80%), trauma management including coagulation management remains an interdisciplinary challenge. While standard conventional coagulation assays are routinely and timely available across all centers, the availability and the access to advanced coagulation assays including functional tests and tests to assess platelet function may differ according to the level of care provided by the centers. Overall, only half of all respondents declared to have access to either functional viscoelastic hemostatic assays (VHAs), e.g. TEG or ROTEM, or to platelet function testing in their local setting while at the same time 44% of the respondents stated the need of advanced technologies for early detection of coagulopathy. This indicates that the clinical use of these advanced technologies has not increased since our survey back in 2014 and although these assays are recommended by the European guideline since 2013 with GRADE 1C [9]. While 83% of the respondents from supraregional trauma centers declared availability and use of VHAs, this was only the case in less than half of all respondents working in regional trauma centers and in only 19% of the respondents working in local centers. Platelet function testing was least used and this even in supraregional trauma centers.

Since 2007, the European guideline on the management of bleeding following major trauma provides evidence-based recommendations on how to diagnose, treat and implement treatment algorithms to manage bleeding trauma patients with regular updates [18]. The current update reemphasizes to initiate monitoring and measures to support coagulation function immediately upon the patient’s admission to the ED [9]. Routine practice shall include the early and repeated monitoring of hemostasis, using either combined CCAs (PT, platelet counts and Clauss fibrinogen level) and/or point-of- care PT/INR (GRADE 1C) and/or VHAs, all with a GRADE 1C recommendation [9]. In contrast to CCAs, VHAs provide faster and more detailed and reliable insights into the quality of the blood clotting disorder thereby allowing a more precision-based and “goal directed” approach in the continuation of treatment [19]. The most recently updated practice management guideline from the Eastern Association for the Surgery of Trauma conditionally recommends using a TEG/ROTEM-based strategy versus non-TEG/ROTEM in adult trauma patients with ongoing hemorrhage and concern for coagulopathy to reduce blood product transfusions and mortality based upon the current literature [20]. Viscoelastic hemostatic assays with corresponding treatment algorithms can rapidly be introduced to facilities initially naïve to this technology [21]. Given the fact that only one open randomised controlled study had been published prior to the 2019 European guideline up-date which supported a survival benefit along with the use of VHA testing it may be that a number of clinicians still questions the benefit of theses assays [9, 22]. This uncertainty may be fueled by the results from the recently published ITACTIC-study that showed no difference in overall outcomes, including mortality and massive transfusion, between VHA and CCT augmented treatment protocols [23]. Although the use of VHAs has repeatedly been associated with less bleeding and fewer blood products transfused, the question to whether timely and targeted management of hemostatic abnormalities after trauma using VHAs may protect against detrimental secondary injury and may translate into improved survival and outcomes remains elusive.

Given the increasing number of elderly trauma patients on preinjury anticoagulant and/or antiplatelet agents and the growing interest in platelet dysfunction after trauma with bleeding and coagulopathy, the current European guideline emphasizes laboratory screening of patients treated or suspected of being treated with anticoagulant and/or antiplatelet agents with a newly introduced GRADE 1C recommendation [9].

While almost all centers regardless of level of care can deliver packed red blood cell concentrates to bleeding trauma patients, there are marked differences in the availability of other blood products and hemostatic agents including coagulation factor concentrates between the centers providing different levels of care. For example, nine out of ten respondents from supraregional trauma centers declared to have access to fibrinogen concentrates, this was only the case in 46% from regional and in 38% from local trauma centers, respectively. Fibrinogen, also referred to as coagulation factor 1, represents the substrate for the clotting process and is the first coagulation factor to reach critical levels in bleeding trauma patients [24, 25]. Low fibrinogen levels upon hospital admission have uniformly been associated with both 24-h and 28-day mortality after trauma [26] and early fibrinogen supplementation could be linked with improved clot stability [27]. At present, the European guideline recommends fibrinogen supplementation with initial 3–4 g if major bleeding is accompanied by viscoelastic signs of a functional fibrinogen deficit or a plasma Clauss fibrinogen level ≤ 1.5 g/L with a GRADE 1C recommendation [9]. Similar differences between different levels of care were noted for other coagulation factors but also for the availability of platelet concentrates. In patients with ongoing hemorrhage, platelet counts should be kept > 100 × 10^9^/l to provide sufficient surface for the blood clotting process [9]. Freeze-dried plasma may be used to compensate non-availability of fresh frozen plasma with easier logistics and in particular regional trauma centers have confirmed its use. Obviously, all trauma centers regardless of level of care have implemented the early use of the antifibrinolytic TXA in bleeding trauma patients based upon the results from the landmark CRASH-2 study despite the fact that follow-up studies could hardly reproduce its results [28, 29]. Potential reasons to why TXA has been widely adopted as major component to almost any pre- and early in-hospital treatment protocol for the severely injured include its widespread availability at cheap cost together with its simple and safe use.

Almost all respondents considered the preinjury use of anticoagulants, and in particular the novel DOACs, as a relevant threat to bleeding trauma patients. Rapid detection of activity and concentration levels is still not widely available 24/7, and this not only in regional or local, but also in supraregional trauma centers. Meanwhile, two specific antidotes have been introduced for the emergency reversal of DOACs, e.g. (1) idarucizumab for direct thrombin inhibitor dabigatran, and (2) andexanet alfa for factor Xa-inhibitors apixaban and rivaroxaban [30]. But even one in three respondents from supraregional trauma centers declared non-access to neither drug in their local setting. Meanwhile, these two agents are cornerstones of revised algorithms to treat bleeding trauma patients, in particular to those with intracranial hemorrhage [31]. Since the approval of both agents in 2015 and 2019, respectively, we have anticipated less clinical issues with preinjury intake of DOACs as compared to our survey from 2014. However, no major changes in responses were found in our present survey. It is expected that andexanet alfa will be adopted by the European guideline during their next revision in 2022. Accordingly, the vast majority of the respondents stressed the need for more improved and timely assessment of patients on preinjury anticoagula therapy and for better access to drug-specific antidotes for emergency reversal, in particular for DOACs.

Although the majority of trauma centers has obviously implemented treatment algorithms for the acute management of bleeding trauma patients with coagulopathy, almost 40% of the respondents admitted inconsistent or non-regular use while at the same time 45% of the respondents stated the need for treatment algorithms. Again, there were differences between centers providing different levels of care. While over 90% of the respondents from supraregional trauma centers declared use of an algorithm, one in three respondents from local trauma centers declared absence of an algorithm in their respective local setting. Meanwhile, several studies have provided a clear signal for outcome improvement for bleeding trauma patients with the implementation and adherence to a treatment algorithm [10–12, 32]. A retrospective study of consecutive bleeding trauma patients and after adjustment for injury severity found that per-patient compliance with protocols based upon the European guideline correlated with decreased mortality at 24 h and at 30 days [32]. Since there was no evolution observed when given results were compared to our 2014 survey, further work is needed to improve adherence to these guidelines with ongoing monitoring to ensure best practice and outcome [14]. Major aspects that differed between the algorithms in place for centers with different levels of care were mostly linked to infrastructure, e.g. the availability for advanced coagulation testing, screening for preinjury use of anticoagulant and/or antiplatelet agents, access to angiographic embolization and to specific blood products and hemostatic agents.

When the results from the present survey were compared to the previous one by our group published back in 2014, it has to be noted that the background of the respondents was slightly different [14]. In the present study, that vast majority of respondents declared to work in the field of trauma/orthopedic surgery (80%) with a higher level of experience in trauma management while mostly working in supraregional trauma centers with higher trauma loads in need for hemostatic therapies. This may be explained by the distribution of the survey through the AUC. In terms of diagnostic approaches, most assessments were used just as often as in 2014 [14]. The use of platelet count had increased from 91 to 100% and functional fibrinogen testing had increased from 44 to 55% compared to our survey from 2014 [14]. Diagnostic approaches to assess preinjury intake of DOACs, e.g. testing for anti-FXa-activity or ECT, had not been approved yet at that time or were not widely available and, therefore, were not queried in 2014. The adherence to a local treatment algorithm in place increased from 61 to 87% compared to our survey from 2014 [14]. Even today, the vast majority of respondents still considers DOACs and platelet inhibitors as a relevant threat to bleeding trauma patients (14). As consequence, timely availability of appropriate testing methods and antidotes against DOACs remain relevant claims forwarded also in the present survey. There was also the claim for more interdisciplinary training programs for rapid clinical control of bleeding, its potential sources and developing coagulopathy.

The major limitation of the present study was the low response rate that could have inflicted a survey bias. This may have had also an impact when comparing the results from the present survey with those from our previous one. A demographic comparison of respondents to non-respondents may have helped to show the representativeness of the sample but this information could not be retrieved from the present survey. However, it has to be acknowledged that the survey was sent out through the AUC to 1875 clinicians all working in DGU^®^-certified supraregional, regional and local trauma centers across Germany either as administrators or deputies. Usually there is one administrator appointed per center with two deputies. This means that a maximum of 3 responses from one center may have been obtained within the given survey. The generalizability and the impact of the given results is limited due to the restriction of the survey to clinicians working in German trauma centers of different levels of care only.

## Conclusion

Our survey confirms differences in infrastructure, logistics and clinical practice management for the detection and treatment of acute trauma hemorrhage and coagulopathy amongst DGU^®^-certified supraregional, regional and local German trauma centers. Further efforts may be undertaken in the context of level of care provided to bleeding trauma patients in order to improve and optimize management and outcomes. Treatment algorithms would profit from a profound basis of randomized controlled trials which may contribute to the reliability of recommendations.

## Supplementary Information

Below is the link to the electronic supplementary material.

Supplementary file1 (PDF 145 KB)

## Declarations

### Availability of data and material

Data can be requested from the corresponding author.
