# Supplementary material for: Infrastructure, logistics and clinical practice management of acute trauma hemorrhage and coagulopathy: a survey across German trauma centers
Source: Eur J Trauma Emerg Surg. 2021 Sep 26;48(6):4461–72. doi: 10.1007/s00068-021-01788-9 (PMC9712372; doi:10.1007/s00068-021-01788-9)
Supplement: Supplementary file 1 — Supplementary file1 (PDF 145 KB) [file 68_2021_1788_MOESM1_ESM.pdf]

Infrastructure, logistics and clinical practice management of acute trauma hemorrhage and coagulopathy: A survey across German trauma centers

European Journal of Trauma and Emergency Surgery

Vivien Karl, Nadine Schäfer, Marc Maegele

Corresponding Author:

Professor Marc Maegele, MD

Institute for Research in Operative Medicine, Faculty of Health, Department of Medicine, Witten/Herdecke University, Ostmerheimer Str. 200, Building 38, D-51109 Cologne, Germany

Department of Traumatology, Orthopaedic Surgery and Sports Traumatology, Cologne-Merheim Medical Centre (CMMC), Witten/Herdecke University, Campus Cologne-Merheim, Ostmerheimer Str. 200, D-51109 Cologne, Germany

E-Mail: Marc.Maegele@t-online.de

1. What is your professional background / your level of experience?

Choose one of the following answers

- ○ Head / Director of Department
- ○ Consultant / Senior Physician / Specialist
- ○ Registrar
- ○ Clinical Assistant / Junior Physician / Staff Grade Doctor

2. What is your speciality?

Choose one of the following answers

- ○ General Surgery
- ○ General Medicine
- ○ Anesthesiology
- ○ Vascular Surgery
- ○ Hematology
- ○ Intensive Care / Critical Care Medicine
- ○ Neurosurgery
- ○ Trauma / Orthopedic Surgery
- ○ Transfusion Medicine
- ○ Accident and Emergency Medicine

○ Other: ____________

3. What is the level of care of the trauma center / hospital you are working in?

Choose one of the following answers

○ Supraregional trauma center (academic university)

○ Supraregional trauma center (non-academic university)

○ Regional trauma center

○ Local trauma center

○ Non-trauma center but participating in trauma care

4. How many severely injured trauma patients (ISS ≥ 16) are treated in your trauma center / hospital each year?

Choose one of the following answers

○ < 16

○ 16 - 25

○ 26 - 50

○ 51 - 100

○ > 100

5. What is the estimated percentage of bleeding trauma patients with coagulopathy and need for hemostatic therapy in your trauma center / hospital?

Choose one of the following answers

○ < 10%

○ 10 - 30%

○ 31 - 50%

○ 51 - 80%

6. Anticoagulants including so-called DOACs (direct oral anticoagulants) and platelet aggregation inhibitors are increasingly being prescribed to prevent secondary ischemic insults. Would you consider the use of these preparations as an increasing risk for this population in the context of uncontrolled or difficult-to-treat trauma-associated bleeding?

Choose one of the following answers

○ NO. I do not see any clinically relevant problems associated with the intake of these agents prior to injury in trauma patients.

○ YES. But the intake of these agents prior to injury causes ONLY MINOR clinical problems.

○ YES. The intake of these agents prior to injury causes MAJOR clinical problems.

7. Which agents would you consider to be the greatest risk in the context of severe and clinically relevant bleeding in trauma patients?

Check any that apply

○ Cumarine derivates (e.g. Marcumar® (phenprocoumon))

○ Heparin

○ Low-molecular weight heparin (z.B. Fragmin® (dalteparin), Clexane® (enoxaparin))

○ Inhibitors of factor Xa (e.g. Xarelto® (rivaroxaban), Eliquis® (apixaban) Lixiana® (edoxaban))

○ Direct thrombin inhibitors (e.g. Pradaxa® (dabigatran), Argatra® (argatroban), hirudin)

○ Antithrombin therapeutics (Anbinex® (antithrombin from human plasma))

○ Acetylsalicylic acid (e.g. Aspirin®)

○ P2Y12-antagonists (e.g. Plavix® (clopidogrel), Ticlid® (ticlopidine), Effient® (prasugrel)

○ Adenosine uptake inhibitors (e.g. Persantin® (dipyridamole), Aggrenox® (dipyridamole & acetylsalicylic acid))

○ Glycoprotein IIb/IIIa inhibitors (e.g. Aggrastat® (tirofiban))

○ Other: ____________

8. Who in your trauma center / hospital is primarily responsible for the initial management of bleeding trauma patients including coagulation management? (Multiple answers possible if a multidisciplinary team applies)

Check any that apply

○ General Surgery

○ General Medicine

○ Anesthesiology

○ Vascular Surgery

○ Hematology

○ Intensive Care/ Critical Care Medicine

○ Neurosurgery

○ Emergency Medicine

○ Trauma / Orthopedic Surgery

○ Transfusion Medicine

○ Other: ____________

9. Which parameters are assessed in your trauma center / hospital in order to evaluate coagulation function disorders after trauma? When are the results of the tests available?

	< 30 min	31-60 min	> 60 min	Not used
Hemoglobin	○	○	○	○
Hematocrit	○	○	○	○
PT/INR/Quick	○	○	○	○
aPTT	○	○	○	○
Platelet count	○	○	○	○
Platelet function (e.g. aggregometry)	○	○	○	○
Fibrinogen (quantitative)	○	○	○	○
Fibrinogen (functional)	○	○	○	○
Viscoelastic tests (TEG/ROTEM)	○	○	○	○
Lactate	○	○	○	○
pH	○	○	○	○

Base excess / deficit	○	○	○	○
Ionised Calcium	○	○	○	○
(Diluted) thrombin time ((d)TT)	○	○	○	○
Anti-FXa-activity	○	○	○	○
Anti-FXa-activity (substance-specific calibrated)	○	○	○	○
FXa (DOAC) plasma concentration	○	○	○	○
Urine test strips (DOAC dipstick)	○	○	○	○
Ecarin clotting time (ECT)	○	○	○	○

10. Which blood products to support coagulation function are available in your trauma center / hospital?

Check any that apply

○ Packed red blood cell concentrate (pRBC)

○ Fresh whole blood (WB)

○ Fresh frozen plasma concentrate (FFP)

○ Thawed fresh plasma

○ Lyophilized plasma / Freeze-dried plasma

○ Platelet concentrate

○ Fibrinogen concentrate

○ Prothrombin complex concentrate (PPSB) (FII; (FVII); FIX; FX, protein C and S)

○ Cryoprecipitate (FVIII, fibrinogen, vWF, FXIII)

○ rFVIIa

○ FXIII-concentrate

○ Further single factor concentrates:

○ Other: ____________

11. What other drugs to support coagulation function are available in your trauma center / hospital?

Check any that apply

○ Tranexamic acid (TXA)

○ Aminocaproic acid

○ Calcium (Ca^{2+})

○ Desmopressin

○ Vitamin K

○ Albumin

○ Andexanet alfa (antidote for FXa inhibitors)

○ Idarucizumab (antidote for dabigatran)

○ Other: ____________

12. Within which time frame after arrival of the bleeding trauma patient is the first blood product administered in your trauma center / hospital?

Choose one of the following answers

○ < 15 min

○ 15 – 30 min

○ 31 – 60 min

○ > 60 min

○ Preclinical administration of blood products possible

13. Has a treatment algorithm (e.g. a mass transfusion protocol) for treating bleeding trauma patients been implemented in your trauma center / hospital?

Choose one of the following answers

○ Yes, regular activation

○ Yes, non-regular activation

○ No

14. What is the rationale behind the treatment algorithm of your trauma center / hospital?

○ International guidelines (e.g. European guideline on management of major bleeding and coagulopathy following trauma)

○ National guidelines (e.g. developed by national societies and / or health authorities)

○ Current literature and clinical-scientific studies

○ Empirical knowledge / local practice

15. What aspects are addressed with your locally implemented treatment algorithm? (Assessment / Investigation / Monitoring)

Check any that apply

○ Clinical evaluation of bleeding and dynamics (ATLS)

○ Risk stratification (e.g. risk assessment for mass transfusion using scores (e.g. TASH score))

○ FAST

○ CT or full-body CT

○ Standard coagulation assays (e.g. PT, INR, Quick, aPTT, fibrinogen, platelet count, etc.)

○ Advanced coagulation monitoring (e.g. viscoelastic tests, aggregometry, multiplate, platelet mapping, etc.)

○ Anticoagulants screening

○ Blood gas analysis

○ Other: ____________

16. What aspects are addressed with your locally implemented treatment algorithm? (Immediate Intervention)

Check any that apply

○ Local bleeding control (compression, tourniquets, pelvic slings)

○ Fluid management (permissive hypotension)

○ Blood pressure management

○ Temperature / acidosis management

○ Coagulants (topical hemostatics)

○ "Damage Control" strategies (e.g. packing, external fixation)

○ Angiographic embolization

○ REBOA (retrograde endovascular balloon occlusion of the aorta)

○ Packed red blood cell concentrates (pRBCs)

○ Fresh frozen plasma concentrates (FFPs)

○ Platelet concentrates

○ Blood products in predefined rations

○ Coagulation factor concentrates

○ Antifibrinolytics (e.g. tranexamic acid)

○ Calcium (Ca^{2+})

○ Other: __________

17. If you were to write a “wish list” in order to improve the early diagnosis and clinical management of bleeding trauma patients with coagulopathy, what would be your choice of priorities?

Check any that apply

○ Implementation of treatment algorithms for the management of bleeding and coagulopathy after trauma

○ Implementation of checklists to guide clinical management

○ Faster availability of test results for standard coagulation

○ Availability of advanced technologies for early detection and monitoring of severe bleeding and coagulopathy after trauma (e.g. viscoelastic tests, aggregometry, multiplate, platelet mapping, etc.)

○ Availability of tests to early detect patients taking direct oral anticoagulants (DOACs) and platelet aggregation inhibitors

○ Timely availability of blood products

○ Timely availability of additional agents / drugs to support coagulation function (e.g. antifibrinolytics, coagulation factor concentrates, etc.)

○ Timely availability of antidotes against DOACs

○ Interdisciplinary training programs to improve clinical skills in early detection and treatment of bleeding and coagulopathy after trauma

○ Improved strategies for faster vertical patient transfers to trauma centers of higher level of care to provide the best possible care for trauma patients

○ No need for improvement
